# Biodiversity of Actinobacteria from the South Pacific and the Assessment of *Streptomyces* Chemical Diversity with Metabolic Profiling

**DOI:** 10.3390/md15090286

**Published:** 2017-09-11

**Authors:** Andrés Cumsille, Agustina Undabarrena, Valentina González, Fernanda Claverías, Claudia Rojas, Beatriz Cámara

**Affiliations:** Laboratorio de Microbiología Molecular y Biotecnología Ambiental, Departamento de Química & Centro de Biotecnología Daniel Alkalay Lowitt, Universidad Técnica Federico Santa María, Valparaíso 2340000, Chile; andres.cumsille@gmail.com (A.C.); agus.undabarrena@gmail.com (A.U.); sekmet.10@gmail.com (V.G.); fclaveriasr@gmail.com (F.C.); rojasf.claudia@gmail.com (C.R.)

**Keywords:** Chilean marine actinobacteria, antimicrobial activity, chemical diversity

## Abstract

Recently, bioprospecting in underexplored habitats has gained enhanced focus, since new taxa of marine actinobacteria can be found, and thus possible new metabolites. Actinobacteria are in the foreground due to their versatile production of secondary metabolites that present various biological activities, such as antibacterials, antitumorals and antifungals. Chilean marine ecosystems remain largely unexplored and may represent an important source for the discovery of bioactive compounds. Various culture conditions to enrich the growth of this phylum were used and 232 bacterial strains were isolated. Comparative analysis of the 16S rRNA gene sequences led to identifying genetic affiliations of 32 genera, belonging to 20 families. This study shows a remarkable culturable diversity of actinobacteria, associated to marine environments along Chile. Furthermore, 30 streptomycete strains were studied to establish their antibacterial activities against five model strains, *Staphylococcus aureus*, *Listeria monocytogenes*, *Salmonella enterica*, *Escherichia coli* and *Pseudomonas aeruginosa*, demonstrating abilities to inhibit bacterial growth of Gram-positive bacteria. To gain insight into their metabolic profiles, crude extracts were submitted to liquid chromatography-high resolution mass spectrometry (LC-HRMS) analysis to assess the selection of streptomycete strains with potentials of producing novel bioactive metabolites. The combined approach allowed for the identification of three streptomycete strains to pursue further investigations. Our Chilean marine actinobacterial culture collection represents an important resource for the bioprospection of novel marine actinomycetes and its metabolites, evidencing their potential as producers of natural bioproducts.

## 1. Introduction

The 2014 World Health Organization Global Report on surveillance of antimicrobial resistance has established that bacterial resistance to commonly used drugs in infection treatments has reached alarming levels in various locations of the world. The occurrence of infectious diseases for which no antibiotic treatment will be available is predicted for the near future [1]. Therefore, there is an urgent need for new bioactive compounds and, despite chemical synthetic efforts, natural products still play a predominant role in drug discovery [2,3]. Taking into account all approved drugs released from 1981 to 2014, approximately 69% of them are either natural products or derived from them [3].

Isolation and culturing of pure bacterial strains and the characterization of their secondary metabolism, remains a significant tool for drug discovery. Many of the products derived from microbial biotechnology that are in current use are obtained from isolated strains [4]. The phylum Actinobacteria represents the most prominent group of microorganisms for the production of bioactive compounds [5,6,7], contributing to nearly 40% of the bioactive secondary metabolite production, and nearly 80% of which are produced by the genus *Streptomyces* [8]. The capability of actinobacterial strains to produce bioactive secondary metabolites relies on their genomic potential, which typically contain a large number of biosynthetic gene clusters, including genes encoding for polyketide synthases (PKS) and non-ribosomal peptide synthetases (NRPS) [9].

The decrease of the number of bioactive compounds isolated from traditional sources such as soil has made critical the investigation of microorganisms from underexplored habitats as a source of novel therapeutic agents [8]. The world’s oceans provide the largest reservoir of microorganisms on earth, and harbor diverse and uncharacterized microbial communities [10]. Marine habitats, due to their ecological pressure, including the competition of space and predation, and physical properties such as salinity, pressure, and temperature variations, have led marine microorganisms to develop unique secondary metabolites with various biological activities to survive in this highly demanding ecosystem [11]. This can be exemplified by marine sediments, which are nutrient rich habitats, harboring a considerable bacterial biodiversity with metabolic and genetic potential to develop bioactive secondary metabolites [12,13]. In addition, many marine microorganisms have developed symbiotic relationships with larger organisms [14]. One example of these associations are marine sponges, where microorganisms could occupy up to 50% of the total sponge biomass volume [15,16,17,18]. Marine sponges are an important source of bioactive compounds, with 243 new compounds reported in the literature in 2013, and 283 in 2014 [19,20]. Studies have reported that sponge-associated microorganisms also produce biologically active secondary metabolites [19,21], suggesting that these microbial communities might play a role in the defense system of the host [15,22,23], therefore, could be the real producers of the sponge-specialized metabolites [19].

In this context, marine actinobacteria are a promising source of novel bioactive compounds, especially from underexplored areas, providing an alternative means for secondary metabolite discovery. The coast of Chile is a favorable ecosystem for this purpose, comprising an extensive coast with a plurality of climates and landscapes. Previous studies have shown the great potential of the coasts of Chile for assessing the culturable actinobacterial diversity indicating the metabolic and genetic potential for the production of bioactive compounds [9,24,25,26]. This study broadens the work on the isolation of marine actinobacteria from marine sediments, sponges and a sea urchin from unexplored regions of both continental and insular Chile ranging from the III to the XI Region, including Easter Island located in Polynesia. Chemical dereplication of streptomycete strains permitted the evaluation of crude extracts derived from our *Streptomyces* collection for the antibiotic production potential through assays against clinically relevant model bacteria.

## 2. Materials and Methods

### 2.1. Marine Sampling

**Figure 1:**
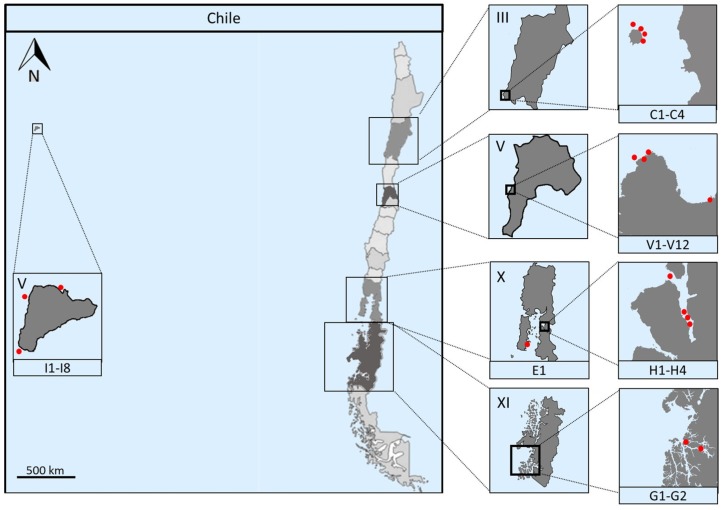
Map of Chile showing the various sampling locations. First insets depict the Chilean regions that were sampled, from top to bottom: Atacama Region (III, samples C1–C4), Valparaíso Region (V, samples V1–V12 and Easter Island, samples I1–I8), Los Lagos Region (X, samples H1–H4) and Aysén del General Carlos Ibáñez del Campo Region (XI, samples G1 and G2). Second insets show the locations sampled in red dots. The Comau fjord in Los Lagos Region and part of the Valparaíso Bay sampling (samples H1–H4 and V7–V12, respectively) have been previously described [24,25]. Scale located in the first inset represents approximately 500 km.

Chile is a long and narrow country, situated on the Pacific coast of South America. It presents an extensive coast in a diverse range of latitudes, comprising a plurality of climates and landscapes that include American, Polynesian and Antarctic territories. The Chilean territory is subdivided into sixteen regions, each one designated by a name and a roman number. Marine samples were collected during six sampling expeditions through distinct coastal regions of Chile (Figure 1). Two marine sediments (samples C1 and C2) and two marine sponges (samples C3 and C4) were collected by scuba divers from Region III, specifically in the surroundings of Chañaral de Aceituno Island. From Region V, two different locations were sampled: Valparaíso bay, where two marine, three beach sediments and one seawater sample (samples V1–V6) were collected, obtaining a total of 12 marine sediments that include previous samples (V7–V12) [25], and from Easter Island, where three marine sediments (samples I1, I4 and I5) and five marine sponges (samples I2, I3, I6, I7 and I8) were obtained. A sea urchin, *Loxechinus albus* (sample E1), was obtained from Chiloé Island located in the X Region, adding to our collection from the Comau fjord (four marine sediments (samples H1–H4), previously studied in our group) [24]; and from the Region XI, specifically from the Penas Gulf, two marine sediments (samples G1 and G2) were kindly handed to our team by Dr. Silva with the Chilean “Cruceros de Investigación Marina en Areas Remotas” program (CIMAR). For information on coordinates and depths of the samples described, see Table S1. All marine and beach sediments, as well as seawater samples were collected dispensing samples directly into sterile 50 mL tubes. Sponge samples were collected by cutting from the sponge with a knife while wearing nitrile gloves. Pieces were put into separate collection bags and brought to surface, maintained at ambient temperature in natural seawater and transported for immediate processing.

### 2.2. Sponge Sample Processing

After sponge collection, each sponge sample was thoroughly rinsed with sterile artificial seawater (ASW) to remove loosely attached bacteria. The rinsed sample was subsequently placed on a sterile surface and a 1 cm^3^ section was cut from the sponge with a sterile scalpel blade. This section of the sponge was placed in a sterile mortar and ground for two minutes, then 1 mL of the liquid obtained after grinding the sponge was transferred to a sterile 15 mL tube and diluted with 9 mL of ASW, obtaining an Aqueous Sponge Extract (ASE) [16].

### 2.3. Isolation of Actinobacteria

#### 2.3.1. Isolation Media

For the isolation of actinobacteria, different media were used (Table 1). Samples C1 and C2 were plated on marine agar (MA) (Difco), ISP2 prepared with ASW, Actinomycete isolation agar, marine sediment, and sea urchin agar. Samples C3 and C4 were plated on MA, ISP2 prepared with ASW, Actinomycete isolation agar, M3 (Peptone, asparagine, sodium propionate, glycerol, K_2_HPO_4_, MgSO_4_, FeSO_4_ and NaCl) [6], and sponge agar [22]. Sea urchin agar was prepared with ASW containing 10 g L^−1^ dry weight of a homogenized filtered solution of gonads and 18 g L^−1^ agar and subsequently autoclaved at 121 °C for 20 min. Marine sediment agar was prepared by a 10% *w*/*v* dilution of marine sediment in ASW and autoclaved at 121 °C for 20 min. The supernatant was collected and completed with ASW to a final volume equal to that of the beginning, then agar (18 g L^−1^) was added and the media is autoclaved again. Marine sediment media were subsequently supplemented with 10 mL L^−1^ of a sterile vitamin solution (biotin 100 mg L^−1^, thiamine 100 mg L^−1^, folic acid 100 mg L^−1^, and nicotinamide 100 mg L^−1^). 

Samples V1–V3 were directly streaked onto Actinomycete Isolation Agar (Difco) prepared with ASW or Milli-Q water. Sample V4 was plated directly into ISP2 prepared with ASW. Samples V5, V6 and E1 were plated on marine sediment and sea urchin agar. Samples I1, I4 and I5 were plated on MA, Actinomycete isolation agar, M1 (Starch, yeast extract and peptone) [6] prepared with ASW, and marine sediment agar. The marine sponge samples, I2, I3, I6, I7 and I8, were plated on the same media as I1, I4 and I5, and additionally on sponge agar. Samples G1 and G2 were plated on MA, Actinomycete isolation agar or ISP2 prepared with ASW.

All marine sponge samples were plated on agar media supplemented with 2% of ASE, with exception of sponge agar, where 10% was used. All isolation media were supplemented with nalidixic acid (25 µg mL^−1^) and cycloheximide (100 µg mL^−1^) as an inhibitor of fast-growing Gram-negative bacteria and fungi, respectively. All plates were incubated up to three months, until visible colonies were observed. The isolation and maintenance of individual colonies were performed as previously described [24,25].

#### 2.3.2. Isolation Methods

Various plating methods were used for the isolation of actinobacteria (Table 1). Sampling techniques for H1–H4 [24] and V7–V12 [25] samples have been previously described by our group. 

Samples C1–C4 were plated directly, serially diluted (10^−4^), used in a stamping technique and a capillary technique. The stamping technique (slightly modified from [6]) consists in drying the samples in a laminar flow hood for 6 h, and then ground with a mortar and pestle, afterwards a sterile falcon tube covered with gauze on the top was pressed into the dried sample and stamped into the surface of the agar eight times in a circular fashion, giving a serial dilution effect. The capillary technique was performed with a glass capillary 20 mm long. 20 µL of a carbon source (glucose or peptone) was added to one end of a capillary, whereas 20 µL of a marine sample (marine sediment or ASE) was added to the opposite end. After 24 h, the glucose or peptone is collected and diluted with 1 mL of sterile water, and plated on agar. Plates were incubated at 20 °C.

Samples V1–V3 were directly streaked and incubated at 4 °C, 20 °C and 30 °C. Sample V4 was directly streaked and incubated at 30 °C. Samples V5, V6 and E1 were either plated directly or went through heat treatment (60 °C, for 60 min) and subsequently incubated at 20 °C or 30 °C. Samples I1–I8 were plated directly, serially diluted (10^−4^), heat treated (60 °C for 60 min) or serially diluted and heat treated (10^−4^, 60 °C for 60 min) and incubated at 20 °C. Samples G1 and G2 were plated directly or went through heat treatment (60 °C for 60 min) and incubated either at 4 °C or 20 °C.

### 2.4. Molecular Identification and Phylogenetic Analysis

An initial screening for the detection of Gram-positive bacteria was used in some samples. The screening consisted of placing one drop of 3% KOH solution on a glass slide. Then a fresh bacterial colony was picked from the surface of a solid media with an inoculation loop and stirred in the KOH solution. After a few seconds of stirring, the inoculation loop was raised to visualize whether the solution was viscous or not. A viscous solution denotes the presence of a Gram-negative bacteria, whereas a non-viscous solution confirms a Gram-positive strain [27]. A PCR assay was performed as a screening for detection of actinobacterial strains using S-C-Act-0235-a-S-20 and S-C-Act-0878-A-19 primers specific for amplification of V3 to V5 regions of 16S rRNA gene from actinobacteria [28]. DNA extraction was prepared as described previously [25]. Each PCR reaction contained 1 µL of genomic DNA, 12.5 µL of GoTaq Green Master Mix (Promega, Madison, WI, USA) and 0.6 µM of each primer in a final reaction volume of 25 µL. The PCR program started with and initial denaturation at 95 °C for 5 min, followed by 35 cycles of DNA denaturation at 95 °C for 1 min, primer annealing at 70 °C for 1 min and extension cycle at 72 °C for 1.5 min, with a final extension at 72 °C for 10 min [24,25]. PCR amplicons were visualized and revealed with SYBR Green staining (E-gel, Invitrogen, Waltham, MA, USA).

A second PCR was performed for positive isolates, using universal primers 27F and 1492R [29]. The reaction contained 1 µL of genomic DNA, 12 µL of GoTaq Green Master Mix (Promega) and 0.2 µM of each primer in a final volume of 25 µL. The reaction started with an initial DNA denaturation at 95 °C for 5 min, followed by 30 cycles of denaturation at 95 °C for 1 min, primer annealing at 55 °C for 1 min and primer-extension at 72 °C for 1.5 min, with a final extension at 72 °C for 10 min. Products were quantified and submitted for purification and sequencing to Macrogen Inc. (Seoul, Korea). For partial sequencing, the universal primer 800R was used, whereas, for the almost complete sequence, the universal primers 27F, 518F, 800R and 1492R were used. Retrieved sequences were manually edited and the genus-level affiliation was validated using the BLAST server from the National Center for Biotechnology Information (NCBI). Sequence alignments were performed using Vector NTI v10 software package (Invitrogen, Waltham, MA, USA). Phylogenetic tree based on the V1 to V9 region of the 16S rRNA gene sequences, was conducted with the program PhyML 3.0, using the 010231 + I + G + F nucleotide substitution model [30] and the maximum likelihood (ML) algorithm with bootstrap values based on 1000 replications [31]. The statistical selection of the nucleotide model substitution was performed with the program jModelTest-2.1 (Posada, Vigo, Spain), using the Akaike information criteria [32]. The tree was visualized using MEGA 6 software [33].

16S rRNA gene sequences of the *Streptomyces* isolates were deposited in GenBank under the following accession numbers: *Streptomyces* sp. CHA1 (MF375002); *Streptomyces* sp. CHA2 (MF375003); *Streptomyces* sp. CHA3 (MF375004); *Streptomyces* sp. CHA15 (MF375005); *Streptomyces* sp. CHA16 (MF375006); *Streptomyces* sp. CHB9 (MF375007); *Streptomyces* sp. CHB19 (MF375008); *Streptomyces* sp. CHC8 (MF375009); *Streptomyces* sp. CHC16 (MF375010); *Streptomyces* sp. CHC141 (MF375011); *Streptomyces* sp. CHD11 (MF375012); *Streptomyces* sp. CHD67 (MF375013); *Streptomyces* sp.Vc67-4 (MF375017); *Streptomyces* sp. Vc17.3-30 (MF375021); *Streptomyces* sp. Vc17.4 (MF375016); *Streptomyces* sp. Vc714c-19 (MF375018); *Streptomyces* sp. Vc74A-19 (MF375019); *Streptomyces* sp. Vc74B-19 (MF375020); *Streptomyces* sp. VB1 (MF375022); *Streptomyces* sp. IpFC-1 (MF375026); *Streptomyces* sp. IpFD-1.1 (MF375023); *Streptomyces* sp. IpFD-6 (MF375024); *Streptomyces* sp. IpHD-1 (MF375025); *Streptomyces* sp. EL5 (MF375014); *Streptomyces* sp. EL9 (MF375015); and *Streptomyces* sp. G11C (MF375027). Other sequences used for constructing the *Streptomyces* phylogenetic tree were reported previously: *Streptomyces* sp. VA42-3 (KM406761), *Streptomyces* sp. VH47-3 (KM406760) and *Streptomyces* sp. VS4-2 (KM406759) in [25]; and *Streptomyces* sp. H-CB3 (KT799851) and *Streptomyces* sp. H-KF8 (KT799850) in [24].

### 2.5. Detection of PKS and NRPS Genes

Amplification of biosynthetic NRPS, PKS type I and PKS type II genes were performed by PCR, using degenerate primers [34,35,36], as recently described [24]. *Streptomyces violeaceoruber* DSM40783 was used as a positive control for all biosynthethic genes and PCR products were visualized in 1% agarose gel electrophoresis, stained with GelRed (Biotium). If amplicons were located at the expected size (700–800 bp for NRPS, 700 bp for PKS type I and 800–900 bp for PKS type II), the detection was determined as positive (+), and negative (--) if the amplicon was absent or present at a different amplicon size.

### 2.6. Antibacterial Activity Screening

All streptomycete strains obtained from the previous studies [24,25] as well as those from this study, with exception of CHD67 strain, were screened for antibacterial activity as previously described [24,25,37], with slight modifications. Four different media were used for growing each isolate: MA, ISP1, ISP2 and TSA, all prepared with ASW [38] with exception of MA. Single colonies of the isolated *Streptomyces* strains were inoculated onto a fresh plate as a middle line dividing the plate into two equal sized halves, and incubated at 30 °C for seven days. Five model bacteria were used to test their susceptibility: *Staphylococcus aureus* NBRC 100910^T^ (STAU); *Listeria monocytogenes* 07PF0776 (LIMO); *Salmonella enterica* subsp *enterica* LT2^T^ (SAEN); *Escherichia coli* FAP1 (ESCO) and *Pseudomonas aeruginosa* DSM 50071^T^ (PSAU). Cultures of the model bacteria grown overnight at 37 °C were used in the streak assay. 10 µL of the model bacteria was placed near the *Streptomyces* line, in the way that they never come in contact with the *Streptomyces* line. For homogeneous seeding, with an inoculation loop, the model strain was seeded perpendicular to the *Streptomyces* line, first toward the border of the plate and subsequently inwards, for a total of five streaks. After inoculation, plates were allowed to dry and then incubated at 37 °C for 24 h. Inhibitions were visualized and ranked as: -, no inhibition; +/-, attenuated growth of the model strain; +, <50% growth inhibition; ++, 50% growth inhibition; and +++, >50% growth inhibition. All experiments were performed in duplicate.

Further antibacterial tests were performed with *Streptomyces* isolates that presented ≥50% inhibition of model bacterial growth or for their isolation source novelty. Selected *Streptomyces* isolates were grown with continuous shaking at 30 °C, in a 50 mL volume of modified ISP1, modified ISP2, modified TSB or MA, depending on which media the antibacterial activity was observed in the previous assays. Crude extracts were obtained at 5, 7 and 10 days of *Streptomyces* growth by solvent extraction using ethyl acetate (EtOAc) in a 1:1 ratio (*v*/*v*) twice. The solvent was almost completely evaporated with speed vacuum, and the remaining extract was dried on a vacuum concentrator. Dried extracts were dissolved in dimethyl sulphoxide (DMSO) (10% *v*/*v*) to the final concentration of 5 µg mL^−1^, and the antibacterial activity was evaluated using 10 µL of extract, over LB agar plates, spread with the bacterial model strains. Plates were incubated for at least 24 h at 37 °C, and inhibition zones were observed. Both 10% DMSO and the medium where the *Streptomyces* strains were grown, were used as negative controls [24].

### 2.7. LC-HRMS Analysis

Crude extracts that showed antimicrobial activity were selected for chemical dereplication using liquid chromatography-high resolution mass spectrometry (LC-HRMS), at 10 mg/mL final concentration. Experiments were carried out as described by de la Cruz [39], with slight modifications, using an HPLC 1200 Rapid Resolution (Agilent, Santa Clara, CA, USA) coupled to a high-resolution mass spectrometer MaXis (Bruker, Billerica, MA, USA). A Zorbax SB-C8 column (Agilent, Santa Clara, CA, USA) was used for separation (2.1 × 30 mm, 3.5 µm) with a constant flow rate of 0.3 mL min^−1^. The mobile phase consisted of: solvent A, water:acetonitrile (AcN) 90:10 and solvent B, water:AcN 10:90; both with ammonium formate 13 mM and 0.01% trifluoroacetic acid (TFA). Gradient composition started with a linear decrease of solvent A from 90% to 0%, and a linear increase of solvent B from 10% to 100%, in six minutes. Then, two minutes maintaining conditions with 0% solvent A and 100% solvent B, followed by recovery of two minutes to attain 90% solvent A and 10% solvent B as initial conditions. Mass spectrometry was operated in positive mode with a spray voltage at 4 kV, 11 L min^−1^ at 200 °C capillary temperature and 280 KPa of pressure at the nebulizer. Absorbance was measured at 210 nm. Molecular formulae and accurate masses were obtained for the predominant components of the crude extract, and comparison of their retention times and masses were used as criteria to search for candidates in the Fundación MEDINA in-house database. Where no match was obtained, a complementary search in the Dictionary of Natural Products of Chapman & Hall database was performed.

LC-HRMS data were additionally analyzed to generate a chemical barcode and to perform a hierarchical cluster analysis. First, the raw Bruker BAF files were converted to a mzXML file with ProteoWizard software (Chambers et al., Nashville, TN, USA) [40], and then imported to the processing software mzMine 2 (Pluskal et al., Okinawa, Japan) [41]. Processing steps were performed as described by Forner [42], with slight modifications. Briefly, mass values were detected using the centroid mode, with a noise level value of 1.5 × 10^3^ counts per second (cps), to generate a list of masses for each scan. Then, a chromatogram was built using each mass generated in the previous step, with a minimal time span of 0.1 min, a *m*/*z* tolerance of 0.005 and a minimal height of 2 × 10^5^ cps. The separated peaks were then deisotoped with a *m*/*z* tolerance of 0.005 and a retention time tolerance of 0.5 min, using the most intense isotope as the representative parent molecule. The peaks in different samples were then aligned, using a *m*/*z* tolerance of 0.01 and a retention time tolerance of 0.2 min. The aligned peaks were filtered to eliminate duplicates, and the files were exported as a comma separated (.CSV) file. Barcoding was manually generated in Microsoft Excel, using the If function (=IF (cell > 0, 1, 0)), creating a binary dataset, where the presence of a certain peak is represented as “1”, shown in black and the absence as “0”, shown in white. Finally, a hierarchical clustering analysis was performed using the Ward’s Method [43] and Squared Euclidean distances.

## 3. Results and Discussion

### 3.1. Biodiversity of Marine Actinobacteria

Several marine samplings were performed throughout the border coast of Chile, including continental and insular territory geographically located in Polynesia, approximately 3700 km inward of the South Pacific basin. These coastal zones are located near the Perú-Chile Trench, where the South America and the Nazca plates converge, creating seismic coupling at shallow depths [44]. A representative map of Chile, depicting sampling locations as red dots is shown in Figure 1. Marine samples were obtained from: III Region, in Chañaral de Aceituno island (samples C1–C4); V Region, comprising Valparaíso coastal border (samples V1–V12) and insular Easter Island (samples I1–I8); X Region, in the Comau fjord, Huinay (samples H1–H4) and in Chiloé Island (E1); and XI Region, within Penas Gulf (Samples G1 and G2). Detailed sampling zones along with coordinates of each specific sampling point are presented in Table S1. Marine sediments and sponges were collected and isolation of actinobacteria was performed, through different selective media and isolation methods. Previous experience from our group revealed that the use of different culture media had a major influence on the isolation of actinobacteria, although there were contrasting results when the same culture media were employed [24,25]. Therefore, in this report, 10 different culture media were tested, and different isolation efficiencies were observed.

**Figure 2:**
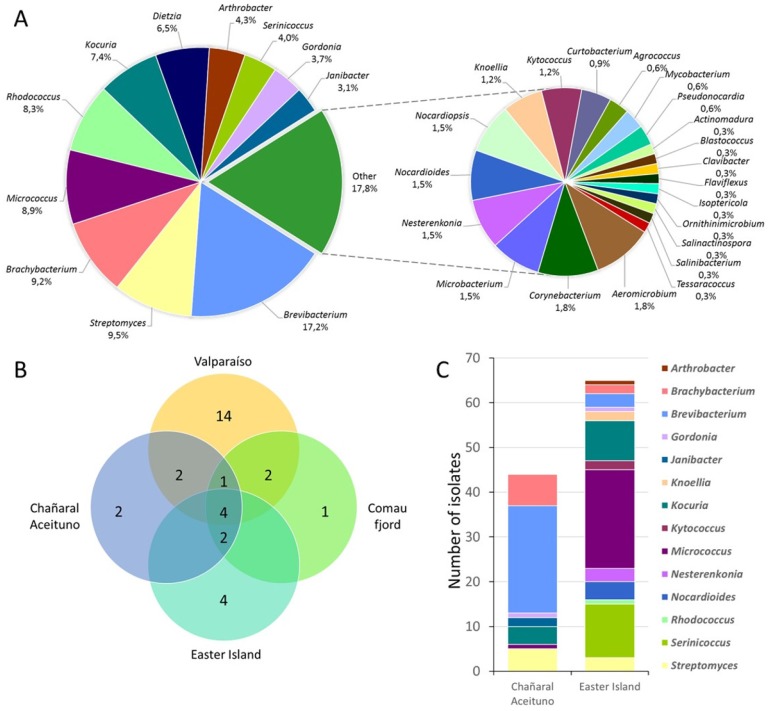
Culturable biodiversity of actinobacteria. (**A**) Pie chart showing respective abundance of the 32 genera isolated throughout all sampling locations. (**B**) Diversity in sediment samples, showing exclusive and shared numbers of genera among Valparaíso, Chañaral Aceituno, Comau fjord and Easter Island samplings. (**C**) Diversity in sponge samples, showing genera abundance between Chañaral de Aceituno and Easter Island samplings.

Identification of the isolates was accomplished by molecular taxonomic methods, by partial 16S rRNA gene sequencing, as previously described [24,25]. Isolates from samples V7–V12 (68 actinobacterial strains) [25] and H1–H4 (25 antibacterial strains) [24] were previously reported by our group. Overall, a total of 325 actinobacterial isolates were retrieved taking into account all sampling locations. From these, 64.9% of the actinobacterial isolates were obtained from 10 marine sediments and 33.5% from seven marine sponge samples, and the remaining were obtained from one sea urchin sample (1.5%). A considerable culturable diversity of actinobacteria was distinguished within Chilean marine samples (Figure 2). Genetic affiliations comprised 32 genera, representing 20 families within the Actinobacteria phylum. Diversity of culturable actinobacteria obtained from all sampling locations along with their relative abundance is shown in Figure 2a. Most abundant isolates were affiliated to the genus *Brevibacterium* (suborder *Micrococcineae*) representing 17.2% of the total diversity, and was followed by *Streptomyces*, *Brachybacterium*, *Micrococcus*, *Rhodococcus*, *Kocuria* and *Dietzia* that varied between 5% and 10% of abundance. The smaller pie chart represents all these genera whose abundance was below 2%, where, interestingly, most of the so-called rare actinobacteria are present (Figure 2a). Rare actinobacteria are those strains that are less likely to be cultivated by conventional methods [45,46]. In addition, usually, the term is used to refer to those strains that are less cultivated than *Streptomyces* strains [47]. In our study, these rare actinobacteria isolates are included within 30 different genera. Furthermore, some genera, such as *Actinomadura*, *Blastococcus*, *Clavibacter*, *Knoellia*, *Kytococcus*, *Nesterenkonia*, *Nocardioides*, *Nocardiopsis*, *Salinibacterium* and *Serinicoccus*, have not been previously described to be present in our Chilean coasts [24,25].

This suggests that Chilean marine environments still represent an underexplored niche to further pursue a viable opportunity for biodiscovery.

Biodiversity of marine sediments from Valparaíso bay, Chañaral de Aceituno Island, Easter Island and Comau fjord samples was analyzed (Figure 2b). For comparison purposes, samples from Penas Gulf were not included in this analysis, as it is currently under development. Four genera were present in all sampling locations, *Brevibacterium*, *Brachybacterium*, *Rhodococcus* and *Streptomyces* (Figure 2b). In addition, unique genera were obtained from each sampling location, demonstrating the value of culturing-dependent techniques. Valparaíso bay demonstrated to present the most notable richness with 14 unique genera (Figure 2b). These isolates belong to *Actinomadura*, *Aeromicrobium*, *Agrococcus*, *Clavibacter*, *Flaviflexus*, *Gordonia*, *Isoptericola*, *Microbacterium*, *Mycobacterium*, *Ornithinimicrobium*, *Pseudonocardia*, *Salinactinospora*, *Salinibacterium*, and *Tessaracoccus* genera, most of them described previously [25]. In addition, four unique genera were retrieved from Easter Island, corresponding to *Kytococcus*, *Knoellia*, *Nesterenkonia* and *Serinicoccus*. These strains were not present in marine sediments from the Chilean continental coastal boarder [24,25], and may represent actinobacteria from insular territory. Gram-positive bacteria such as actinobacteria, are more commonly observed in organic-rich habitats like sediments within the marine environment [48]. Given that organic content is variable among locations, this may explain the observed heterogeneous biodiversity [49]. Nevertheless, actinobacteria have been successfully isolated from numerous marine sediment samples in different environments [6,12,50,51,52,53,54,55,56].

On the other hand, biodiversity of sponge-derived samples was compared, comprising samples from Chañaral de Aceituno Island and Easter Island (Figure 2c). Respective genera abundance showed to be considerably different between both sampling sites. From Chañaral de Aceituno Island, a total of 44 actinobacterial strains were retrieved, where *Brevibacterium* and *Brachybacterium* represented the major abundance, with 54.5% and 15.9%, respectively (Figure 2c). As for Easter Island samples, *Micrococcus* was the most abundant genus (33.8%), followed by *Serinicoccus* with 18.5% and *Kocuria* with 13.8%, among a total of 65 strains (Figure 2c). Sponge-derived actinobacteria, with antimicrobial activity, have been described from several locations worldwide, such as the Mediterranean Sea [37,57,58]; Australia’s Great Barrier Reef [59]; Conch Reef in Florida [16]; the Caribbean Sea of Puerto Rico [14]; South [60,61] and Northwest [62] China Sea; North Java Sea in Indonesia [63]; the Red Sea [64]; and the Baltic Sea [65,66]. To our knowledge, this is the first report dealing with the isolation of actinobacteria from marine sponges of the Chilean coast.

For further experiments, only streptomycete strains were selected. Overall, a total of 31 *Streptomyces* were obtained from all sampling locations, accounting for 8.5% of total actinobacterial abundance in this study (Figure 2a). From these, 67.7% and 25.8% of the strains were recovered from sediment and sponge samples, respectively. Although *Streptomyces* strains were widely distributed in all Chilean marine sampling locations (Figure 2b), the majority of strains were provided by a sediment sample from Chañaral de Aceituno Island (22.5% of *Streptomyces*), followed by a sediment sample from Valparaíso bay (19.3% of *Streptomyces*). Therefore, the antimicrobial potential of our Chilean marine *Streptomyces* collection was explored using an integrative approach, which involved phylogenetic and chemical dereplication in order to gain insights into their metabolic profile.

### 3.2. Phylogenetic Analysis of Marine Streptomyces and Presence of Biosynthetic Genes

**Figure 3:**
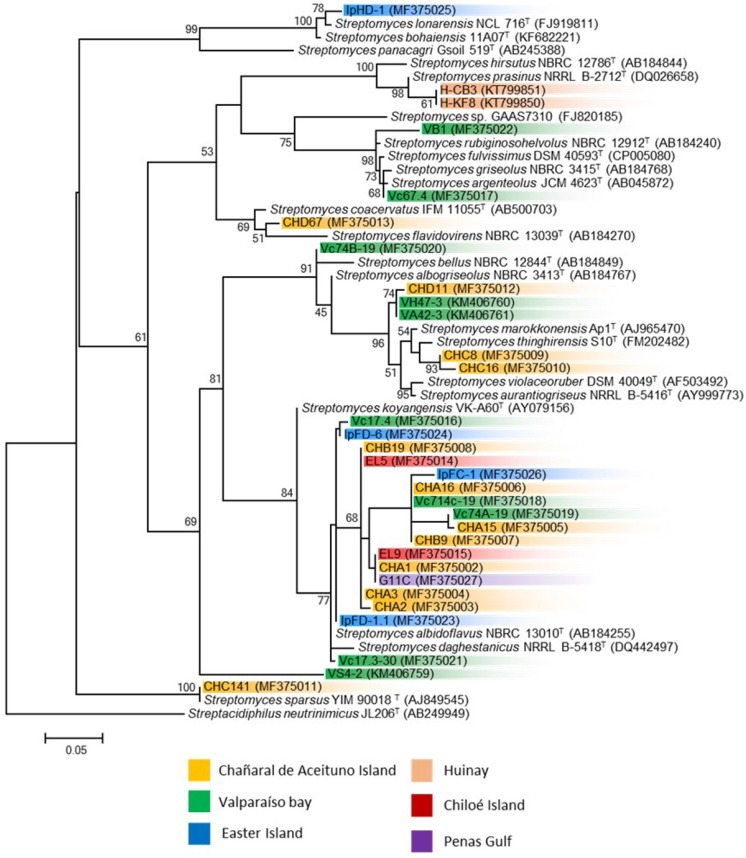
Phylogenetic tree of representative *Streptomyces* isolated along the coasts of Chile. Phylogenetic tree, based on the V1 to V9 region of the 16S rRNA gene sequences, was conducted with the PhyML 3.0 using the maximum likelihood algorithm with bootstrap values based on 1000 replications. The statistical selection of the nucleotide model substitution was performed with jModelTest-2.1, supporting the proposed branching order shown at consistent nodes (values below 50% not shown). Gene sequence positions 101–1395 were considered, according to the *Escherichia coli* K12 (AP012306) 16S rRNA gene sequence numbering. Outgroup is defined as *Streptacidiphilus neutrinimicus* JL206T. GenBank accession numbers of 16S rRNA sequences are given in parentheses. Scale bar corresponds to 0.05 substitutions per nucleotide positions.

Almost complete sequencing of the 16S rRNA gene was performed to 31 streptomycete strains isolated from these sediment, sponges and sea urchin samples. Comparison of the V1 to V9 region of the 16S rRNA gene sequences (between 1327 and 1471 nucleotides) of the 31 strains was used to construct a phylogenetic tree. Thirty of the thirty-one strains shared 99.1–99.9% sequence similarities with a closest type strain (Table 2). The phylogenetic analysis presented implies a diversity of culturable streptomycetes within marine samples derived from various latitudes of the Chilean coast. From the different clades observed in the phylogenetic tree constructed by the maximum likelihood algorithm (Figure 3), there is not a clear group pattern considering the sampling sites and sample types. Notably, one defined clade is conformed with 55% of the *Streptomyces* strains, comprising strains from all sampling sites, with exception of strains obtained from Huinay, in the Comau fjord [24]. The closest type strain to this group of *Streptomyces* is *S. albidoflavus* NBRC 13010^T^ (AB184255). *S. albidoflavus* strains are known to be ubiquitous in nature, and have been widely isolated from many diverse environments, comprising terrestrial lichens, marine microalgae, deep-sea ecosystems, marine invertebrates and repeatedly isolated from atmospheric precipitation [67]. This is in agreement with our findings, where four of the five locations sampled in this study have retrieved strains belonging to this *S. albidoflavus* clade.

Three strains form a different clade each, showed differences with other isolated *Streptomyces* strains. Strain IpHD-1 is the only streptomycete isolated from marine sediments derived from Easter Island, which could explain the difference observed when compared to other isolated strains (Figure 3). Strain VS4-2, previously reported [25], is the only strain presenting less than 99% similarity when compared with closely related type strains, which is in agreement with the phylogenetic analysis where it forms a single individual branch. Strain CHC141 is one of the five *Streptomyces* isolated from a marine sponge from Chañaral de Aceituno Island.

The presence of biosynthetic PKS (type I and II) and NRPS genes were detected by PCR in all *Streptomyces* strains (Table 2). Most isolates showed the presence of at least one type of biosynthetic gene. Among them, NRPS was the predominant gene observed (77%), followed by PKS type II (32%) and PKS type I (26%).

### 3.3. Antimicrobial Potential of Marine Streptomyces

All *Streptomyces* strains, with exception of strain CHD67, were evaluated for antibacterial activity using the cross-streak method [37], as previously described [24,25]. Strains were tested in four different culture media, against the following model bacteria: *Staphylococcus aureus* (STAU), *Listeria monocytogenes* (LIMO), *Pseudomonas aeruginosa* (PSAU), *Escherichia coli* (ESCO) and *Salmonella enterica* (SAEN). In total, 185 (31%) growth inhibitions were detected out of 600 total interactions tested (Table 3), from which 28 out of 30 (93%) *Streptomyces* strains showed inhibition against at least one model bacterium. Inhibitions were observed more frequently against Gram-positive bacteria where 26 (87%) of the tested streptomycete strains presented inhibitions against STAU and the same amount of strains presented inhibition against LIMO in at least one of the four media tested, followed by ESCO with 23 strains (77%), SAEN, with 10 (33%) and PSAU with only nine strains (30%). This could be because the outer membrane of the Gram-negative bacteria could serve as a barrier for protection against toxic compounds, including antibiotics [68,69], making these strains more resistant than Gram-positive bacteria.

Considering all the media used for this screening, inhibitions in ISP2 were generally more active against STAU, LIMO, PSAU and SAEN than ESCO; although glucose has been reported to interfere with antibiotic production, even at low concentrations as in these media. The difference observed in the activities among different culture media against the same model strain, could be due to the carbon source which regulates the antibiotic production, subsequently one strain could produce several secondary metabolites by changing the growth conditions [2,70,71,72], and the activities observed in different media could be due to different compounds produced in each medium [71].

Of the *Streptomyces* strains showing inhibition in the cross-streak method, 14 strains were selected either for presenting ≥50% inhibition against at least one model strain (strains CHA3, CHC8, CHC16, Vc67-4, Vc74A-19, VB1, IpHD-1, H-CB3, H-KF8 and G11C), or for their origin novelty (CHD11, IpFC-1, EL9, and VH47-3), to further evaluate the antimicrobial activity against the same five model bacteria of their EtOAc crude extracts from liquid cultures (Table 4). All of these strains, with exception of strain VB1 showed the presence, by PCR, of at least one biosynthetic gene. Of the 14 selected strains, ten of them (71%) maintained at least part of the activity previously observed in the cross-streak assay. All growth inhibitions observed in the cross-streak assay against Gram-negative bacteria, were not subsequently observed with crude extracts. This difference could be caused by a nutrient depletion, which is responsible of some false positives in the cross-streak assay [73]. However, other possibilities, such as variations in growth conditions and chemical affinity to the solvent used for extraction, can not be ruled out [70,74]. On the other hand, in the case of strains CHD11, VH47-3, Vc67-4 and G11C, their EtOAc crude extracts were active against STAU, while this activity was not observed in the cross-streak assay.

### 3.4. Chemical Profiling of Selected Marine Streptomyces

**Figure 4:**
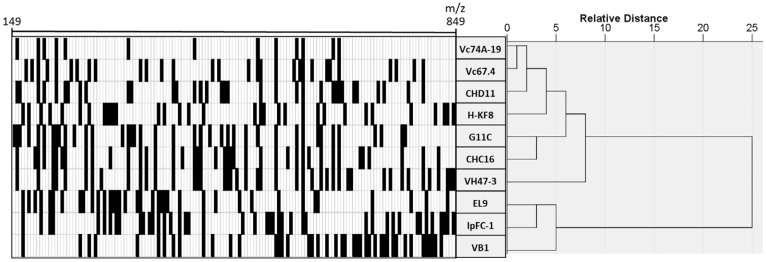
Chemical barcoding and hierarchical cluster analysis based on liquid chromatography-high resolution mass spectrometry metabolic profiles from selected *Streptomyces* strains. Retention times and *m*/*z* values were used as variables. Vertical axis depicts *Streptomyces* strains and horizontal axis shows variables that represent a single compound, and its presence is represented as a black square.

Our final aim was to prioritize strains for further studies considering their metabolic diversity. To display the metabolic profile of the EtOAc extracts from selected *Streptomyces*, the LC-HRMS data were analyzed to detect the different metabolites, comprising *m*/*z* values from 149 to 849. The data were then normalized as a binary code, where “1” (shown as a black square) shows the presence of certain metabolite in one sample and “0” (shown as a white square) the absence. To assess the relative distance of each metabolic profile, a hierarchical clustering was performed (Figure 4). The hierarchical cluster analysis shows that the strains can be roughly grouped into two distinct clusters according to their chemical profiles. One of these groups is comprised by three strains: EL9, IpFC-1 and VB1. However, strains VB1 and VH47-3 branch separately compared to the rest of the strains, suggesting differences in their respective metabolic profiles in comparison to the other strains analyzed.

Dereplication is a relatively fast means for the identification of known chemical entities, notably aiding the quest for the search for novel antimicrobial compounds. Dereplication studies are useful as a tool for, in our case, selecting the streptomycete strains that have potentially new chemical entities and therefore worthwhile for subsequent fractioning, demonstrating activity and ultimately structure elucidation with NMR studies. According to Fundación Medina LC-HRMS report, all strains submitted to LC-HRMS showed the presence of compounds that have similar feature of the UV spectra of various diketopiperazines. These cyclic dipeptides have been previously obtained from marine actinomycetes isolated from sediments in Fiji, Yellow River estuary and Huanghai Beach in China [75]. Some of the diketopiperazines such as Cyclo(Trp-Pro), Cyclo(Phe-Pro) and Cyclo(Leu-Pro) have been shown to exhibit antibacterial activity against Gram-positive and Gram-negative bacteria. Nevertheless, since they are abundantly present in all extracts evaluated, these molecules are more likely to play a role as signal molecules. Another family of compounds presumably present in extracts derived from strain VB1 culture was anthraquinones. Baumycins A1 and A2 have been previously isolated from *Streptomyces*, showing antimicrobial activity against Gram-positive bacteria [76]. Benastatin metabolites also have been isolated from *Streptomyces*, and benastatins A and B have shown activity against Gram-positive bacteria, on the contrary, there were no report of biological activity of benastatin J (compound putatively produced by *Streptomyces* sp. VB1) [77]. Surugamides are cyclic octapeptides obtained from marine *Streptomyces* isolated in Suruga Bay, Japan [78]. Surugamide A has a moderate antibacterial activity against STAU [79] and has the same planar structure as champacyclins, differing in two amino acid residues. This compound may be putatively identified in extracts obtained from strains IpFC-1, EL9 and G11C, whereas Surugamide B, C, D or E may be putatively identified in extracts of strains IpFC-1 and G11C. Most interestingly, our dereplication results showed that strains VB1, VH47-3 and Vc74A-19 have compounds with chemical formulae not identified in the Fundación Medina database.

Considering all of the datasets obtained in this study, that is, bioactivity of crude extracts, phylogenetic analysis of the strains, metabolic profiling and dereplication, the next step was to integrate the information to guide the prioritization of strains to be further analyzed. This is a critical step for drug discovery since chemical analysis of secondary metabolites can be very time-consuming, labor-intensive, and costly as well as can easily end with the high risk of isolating known antibacterial compounds. Strains VB1 and Vc67.4, both isolated from Valparaíso Bay, are grouped within the same clade when observed in the 16S rRNA gene phylogenetic tree (Figure 3), however they cluster notably differently when their metabolic profiles were compared (Figure 4). This could suggest that the bacterial extracts of these strains could have chemically distinct metabolites [80], although both bacterial extracts have bioactivity against two model Gram-positive bacteria (Table 4). In the same extent, strains CHD11 and VH47-3 cluster relatively close when compared by 16S rRNA gene sequence, but appear distinct when their metabolic profiles were observed. The latter is in agreement with their bioactivity profiles, where strain CHD11 is active against one of the five model bacteria, while strain VH47-3 inhibits the growth of two model bacteria. Of the four *S. albidoflavus* like strains with metabolic profiles presented in this work, strains EL9 and IpFC-1 cluster together, therefore are considered to have similar metabolic profiles. In addition, both of these strains are likely to produce a compound that was tentatively assigned as surugamide A by the dereplication studies.

## 4. Conclusions

In this study, actinobacteria from marine sediments and sponges of five locations along various latitudes of the coast of Chile (Chañaral de Aceituno island, III Region; Valparaiso Bay and Easter Island, V Region; Comau fjord, X Region; and Penas Gulf, XI Region) were isolated showing an overall striking diversity represented by 32 genera. Chemical dereplication strategy provided possibilities to putatively identify chemical entities in each of the bioactive extracts, as well as determining the amount of unknown compounds. By combining various criteria such as phylogenetic designations, bioactivity screening, metabolic profiling and dereplication of selected streptomycete strains, we were able to pinpoint three strains, VB1, VH47-3 and Vc74A-19, considered relevant for further scaling-up studies, suggesting the possibility that bioactive compounds produced by these strains could have possibilities for being novel.

A small subset of marine-derived *Streptomyces* strains was used for metabolic profiling to see whether we could prioritize strains for further chemical studies. This study gives us an overview to follow up metabolic profiling analysis and dereplication studies for the other genera, such as the rare actinobacteria isolated in this study.

## Figures and Tables

**Table 1:** Samples treatments and cultivation media for the isolation of Actinobacteria.

Sample	Sampling Site	Isolation Method	Isolation Media	Temperature
**C1**	Chañaral de Aceituno Island	Direct serially diluted (10^ࢤ4^) Stamping technique capillary technique	MA ISP2 prepared with ASW actinomycete isolation agar sea urchin agar marine sediment agar	20 °C
**C2**				
**C3**			MA ISP2 prepared with ASW actinomycete isolation agar M3 sponge agar	
**C4**				
**V1**	Valparaíso	Direct	Actinomycete isolation agar actinomycete isolation agar prepared with ASW	4 °C 20 °C 30 °C
**V2**				
**V3**				
**V4**		Direct	ISP2 prepared with ASW	30 °C
**V5**		Direct heat treatment	Sea urchin agar marine sediment agar	20 °C 30 °C
**V6**				
**I1**	Easter Island	Direct serially diluted (10^−4^) heat treatment serially diluted (10^−4^) with heat treatment	MA actinomycete isolation agar M1 prepared with ASW marine sediment agar	20 °C
**I2**			MA actinomycete isolation agar M1 prepared with ASW marine sediment agar sponge agar	
**I3**				
**I4**			MA actinomycete isolation agar M1 prepared with ASW marine sediment agar	
**I5**				
**I6**			MA actinomycete isolation agar M1 prepared with ASW marine sediment agar sponge agar	
**I7**				
**I8**				
**E1**	Chiloé Island	Direct heat treatment	Sea urchin agar marine sediment agar	20 °C 30 °C
**G1**	Penas Gulf	Direct heat treatment	MA actinomycete isolation agar ISP2 prepared with ASW	4 °C 20 °C
**G2**				

**Table 2:** Characteristics of *Streptomyces* strains isolated from the South Pacific.

Strain	Closest Type Strain (Accession Number) (% Identity)	Sample Information		Biosynthetic Genes			Reference
		Sampling Site	Sample Type	PKS I	PKS II	NRPS	
CHA1	*S. albidoflavus* NBRC 13010^T^ (AB184255) (99.51)	Chañaral de Aceituno Island	Marine sediment	-	-	+	This study
CHA2	*S. albidoflavus* NBRC 13010^T^ (AB184255) (99.23)			-	-	+	This study
CHA3	*S. albidoflavus* NBRC 13010^T^ (AB184255) (99.65)			-	+	+	This study
CHA15	*S. albidoflavus* NBRC 13010^T^ (AB184255) (99.40)			-	+	-	This study
CHA16	*S. albidoflavus* NBRC 13010^T^ (AB184255) (99.62)			-	+	+	This study
CHB9	*S. albidoflavus* NBRC 13010^T^ (AB184255) (99.65)			-	-	+	This study
CHB19	*S. albidoflavus* NBRC 13010^T^ (AB184255) (99.44)			-	-	+	This study
CHC8	*S. thinghirensis* S10^T^ (FM202482) (99.59)		Marine sponge	-	-	+	This study
CHC16	*S. thinghirensis* S10^T^ (FM202482) (99.52)			-	-	+	This study
CHC141	*S.* *sparsus* YIM 90018^T^ (AJ849545) (100)			+	+	+	This study
CHD11	*S.* *aurantiogriseus* NRRL B-5416^T^ (AY999773) (99.38)			+	+	+	This study
CHD67	*S.* *coacervatus* AS-0823^T^ (AB500703) (99.59)			+	-	+	This study
VA42-3	*S. aurantiogriseus* NRRL B-5416^T^ (AY999773) (99.37)	Valparaíso	Marine sediment	-	-	+	[25]
VH47-3	*S. aurantiogriseus* NRRL B-5416^T^ (AY999773) (99.37)			-	-	+	[25]
VS4-2	*S. fabae* T66^T^ (KM229360) (98.32)			-	+	-	[25]
Vc17.3-30	*S. albidoflavus* NBRC 13010^T^ (AB184255) (99.93)			-	-	-	This study
Vc17.4	*S. exfoliatus* NBRC 13475^T^ (AB184868) (99.79)			-	-	+	This study
Vc67-4	*S. argenteolus* AS 4.1693^T^ (D44272) (99.93)			+	+	+	This study
Vc714c-19	*S. albidoflavus* NBRC 13010^T^ (AB184255) (99.59)			-	-	+	This study
Vc74A-19	*S. albidoflavus* NBRC 13010^T^ (AB184255) (99.45)			-	-	+	This study
Vc74B-19	*S. albogriseolus* NBRC 3413^T^ (AB184315) (99.72)			-	-	-	This study
VB1	*S. pratensis* ch24^T^ (JQ824046) (99.86)			-	-	-	This study
IpFC-1	*S. albidoflavus* NBRC 13010^T^ (AB184255) (99.50)	Easter Island	Marine sponge	+	-	+	This study
IpFD-1.1	*S. albidoflavus* NBRC 13010^T^ (AB184255) (99.93)			+	-	+	This study
IpFD-6	*S. albidoflavus* NBRC 13010^T^ (AB184255) (99.79)			+	+	+	This study
IpHD-1	*S. lonarensis* NCL 716^T^ (FJ919811) (99.73)		Marine sediment	+	-	+	This study
EL5	*S. albidoflavus* NBRC 13010^T^ (AB184255) (99.65)	Chiloé Island	Sea Urchin	-	-	-	This study
EL9	*S. albidoflavus* NBRC 13010^T^ (AB184255) (99.71)			-	+	-	This study
H-CB3	*S. prasinus* NRRL B-2712^T^ (DQ026658) (99.86)	Huinay	Marine sediment	-	-	+	[24]
H-KF8	*S. prasinus* NRRL B-2712^T^ (DQ026658) (99.93)			-	+	+	[24]
G11C	*S. albidoflavus* NBRC 13010^T^ (AB184255) (99.64)	Penas Gulf	Marine sediment	-	-	+	This study

+ indicates amplicon is present at the expected size, - indicates the absence or the presence of an amplicon at a different expected size.

**Table 3:** Antibacterial activity of *Streptomyces* strains against model bacteria using cross-streak method.

Strains	STAU				LIMO				PSAU				SAEN				ESCO			
	TSA	MA	ISP1	ISP2	TSA	MA	ISP1	ISP2	TSA	MA	ISP1	ISP2	TSA	MA	ISP1	ISP2	TSA	MA	ISP1	ISP2
**CHA1**	-	+/--	+/-	+	-	+/-	-	-	-	-	-	-	-	-	-	-	+	-	-	-
**CHA2**	--	+/--	-	+	-	+/-	-	+	-	-	-	-	-	-	-	-	+	-	-	-
**CHA3**	--	--	-	+	-	-	-	+	-	-	-	-	-	-	-	-	++	-	-	-
**CHA15**	--	+/--	+/-	+	-	+/-	-	+/-	-	-	-	-	-	-	-	-	+	-	-	-
**CHA16**	--	+/--	+/-	+	-	+/-	+/-	+/-	-	-	-	-	-	-	-	-	+	-	-	-
**CHB9**	--	+/--	+/-	+	-	+/-	+/-	-	-	-	-	-	-	-	-	-	+	-	-	-
**CHB19**	--	+/--	+/-	+	-	+/-	+/-	+	-	-	-	-	-	-	-	-	+	-	-	-
**CHC8**	+	+++	+++	+	-	+	-	-	-	+	-	-	-	++	-	-	+	+++	+++	-
**CHC16**	+++	+	+++	+	-	-	-	-	+/-	-	-	-	+	+	-	-	++	+	+++	-
**CHC141**	--	--	-	-	-	-	-	-	-	-	-	-	-	-	-	-	-	-	-	-
**CHD11**	--	--	-	-	-	-	-	-	-	-	-	-	-	-	-	-	-	-	-	-
**VA42-3**	--	--	-	-	-	-	-	-	-	-	-	-	-	-	-	+/-	-	-	-	-
**VH47-3**	--	--	-	-	-	-	-	+	-	-	-	-	-	-	-	+/-	-	-	-	-
**VS4-2**	--	+/--	-	+/-	-	+	-	+/-	-	-	-	-	-	-	-	+/-	-	-	-	+/-
**Vc17.3-30**	--	+/--	-	+	-	+/-	-	-	-	-	-	-	-	-	-	-	+/-	-	-	+/-
**Vc17.4**	+/--	+/--	-	-	-	+/-	-	-	-	-	-	-	-	-	-	-	-	-	-	-
**Vc67-4**	+/--	--	+/-	+++	-	-	+/-	+++	-	-	-	+/-	+/-	-	-	+/-	-	-	-	+/-
**Vc714c-19**	--	+/-	-	+	-	+/-	-	+	-	-	-	-	-	-	-	-	-	-	-	-
**Vc74A-19**	+/--	-	-	-	-	-	-	+++	-	-	-	-	-	-	-	-	+/-	-	-	-
**Vc74B-19**	--	-	-	+	-	-	-	+/-	-	-	-	-	-	-	-	-	-	-	-	-
**VB1**	+++	++	+	+++	+++	+++	++	+++	++	-	-	-	-	+/-	-	-	+++	-	+/-	-
**IpFC-1**	+/--	+	+/-	+	-	-	-	+/-	-	-	-	-	-	-	-	-	+++	-	-	-
**IpFD-1.1**	--	+	+/-	+	-	+/-	-	-	-	-	-	-	-	-	-	-	+++	-	-	-
**IpFD-6**	+/--	+	+/-	+/-	-	-	+/-	+/-	-	-	-	-	-	-	-	-	++	-	-	-
**IpHD-1**	--	-	-	++	-	+/-	-	+/-	-	-	-	-	-	-	-	-	++	-	-	-
**EL5**	+	+/-	+/-	+	+/-	+/-	+/-	+/-	+/-	-	-	+/-	-	-	-	-	+/-	-	-	+/-
**EL9**	+	+/-	+/-	+	-	-	+/-	+/-	-	-	-	+/-	+/-	-	-	-	+	-	-	-
**H-CB3**	+++	+++	+++	+++	+/-	+++	+++	+/-	-	-	-	+/-	-	-	+++	-	++	++	+++	+
**H-KF8**	+++	+++	+++	+++	+/-	+++	+++	+/-	-	-	-	+/-	-	-	+++	-	+	++	+++	+
**G11C**	--	+/-	+/-	+	-	+/-	-	+/-	+++	-	-	-	-	-	-	-	+++	-	++	-

--, no inhibition; -+/-, attenuated growth; +, <50% growth inhibition; ++, 50% growth inhibition; +++, >50% growth inhibition in the cross-streak assay. All media were prepared with ASW, with exception of MA.

**Table 4:** Antibacterial activity of selected *Streptomyces* strains against model bacteria using EtOAc crude extracts.

*Streptomyces* Strain	Model Bacteria				
	STAU	LIMO	PSAU	SAEN	ESCO
CHC16	+	-	-	-	-
CHC8	-	-	-	-	-
CHA3	-	-	-	-	-
CHD11	+	-	-	-	-
EL9	+	-	-	-	-
H-CB3	-	-	-	-	-
H-KF8	+	-	-	-	-
VH47-3	+	+	-	-	-
Vc67-4	+	+	-	-	-
Vc74A-19	+	-	-	-	-
VB1	+	+	-	-	-
IpFC-1	+	-	-	-	-
IpHD-1	-	-	-	-	-
G11C	+	-	-	-	-

## Supplementary Materials

The following are available online at www.mdpi.com/1660-3397/15/9/286/s1, Table S1. Characteristics of the marine samples used for isolation of Actinobacteria in this study.

## References

[1] World Health Organization (WHO) (2014). Antimicrobial Resistance.

[2] Genilloud O. (2014). The re-emerging role of microbial natural products in antibiotic discovery. Antonie Van Leeuwenhoek.

[3] Newman D.J., Cragg G.M. (2016). Natural products as sources of new drugs from 1981 to 2014. J. Nat. Prod..

[4] Joint I., Mühling M., Querellou J. (2010). Culturing marine bacteria—An essential prerequisite for biodiscovery: Minireview. Microb. Biotechnol..

[5] Williams P.G. (2009). Panning for chemical gold: Marine bacteria as a source of new therapeutics. Trends Biotechnol..

[6] Mincer T.J., Jensen P.R., Kauffman C.A., Fenical W. (2002). Widespread and persistent populations of a major new marine actinomycete taxon in ocean sediments. Society.

[7] Prieto A., Villarreal L., Forschner S., Bull A., Stach J., Smith D., Rowley D., Jensen P. (2014). Targeted search for actinomycetes from near-shore and deep sea marine sediments. FEMS.

[8] Bull A.T., Stach J.E., Ward A.C., Goodfellow M. (2005). Marine actinobacteria: Perspectives, challenges, future directions. Antonie Van Leeuwenhoek.

[9] Undabarrena A., Ugalde J.A., Seeger M., Cámara B. (2017). Genomic data mining of the marine actinobacteria *Streptomyces* sp. H-KF8 unveils insights into multi-stress related genes and metabolic pathways involved in antimicrobial synthesis. PeerJ.

[10] Dalisay D.S., Williams D.E., Wang X.L., Centko R., Chen J., Raymond J. (2013). Marine sediment-derived *Streptomyces* bacteria from British Columbia, Canada are a promising microbiota resource for the discovery of antimicrobial natural products. PLoS ONE.

[11] Donia M., Hamann M.T. (2003). Marine natural products and their potential applications as anti-infective agents. Lancet Infect. Dis..

[12] Duncan K., Haltli B., Gill K.A., Kerr R.G. (2014). Bioprospecting from marine sediments of New Brunswick, Canada: Exploring the relationship between total bacterial diversity and actinobacteria diversity. Mar. Drugs.

[13] Penesyan A., Kjelleberg S., Egan S. (2010). Development of novel drugs from marine surface associated microorganisms. Mar. Drugs.

[14] Vicente J., Stewart A., Song B., Hill R.T., Wright J.L. (2013). Biodiversity of actinomycetes associated with caribbean sponges and their potential for natural product discovery. Mar. Biotechnol..

[15] Graça A.P., Bondoso J., Gaspar H., Xavier J.R., Monteiro M.C., De La Cruz M., Oves-Costales D., Vicente F., Lage O.M. (2013). Antimicrobial activity of heterotrophic bacterial communities from the marine sponge *Erylus discophorus* (Astrophorida, Geodiidae). PLoS ONE.

[16] Montalvo N.F., Mohamed N.M., Enticknap J.J., Hill R.T. (2005). Novel actinobacteria from marine sponges. Antonie Van Leeuwenhoek.

[17] Thomas T.R.A., Kavlekar D.P., LokaBharathi P.A. (2010). Marine drugs from sponge-microbe association—A review. Mar. Drugs.

[18] Khan S.T., Musarrat J., Alkhedhairy A.A., Kazuo S. (2014). Diversity of bacteria and polyketide synthase associated with marine sponge *Haliclona* sp. Ann. Microbiol..

[19] Blunt J.W., Copp B.R., Munro M.H.G., Northcote P.T., Prinsep M.R. (2016). Marine natural products. Nat. Prod. Rep..

[20] Blunt J.W., Copp B.R., Munro M.H.G., Northcote P.T., Prinsep M.R. (2015). Marine natural products. Nat. Prod. Rep..

[21] Graça A.P., Viana F., Bondoso J., Correia M.I., Gomes L., Humanes M., Reis A., Xavier J.R., Gaspar H., Lage O.M. (2015). The antimicrobial activity of heterotrophic bacteria isolated from the marine sponge *Erylus deficiens* (Astrophorida, Geodiidae). Front. Microbiol..

[22] Selvin J., Joseph S., Asha K.R.T., Manjusha W.A., Sangeetha V.S., Jayaseema D.M., Antony M.C., Denslin Vinitha A.J. (2004). Antibacterial potential of antagonistic *Streptomyces* sp. isolated from marine sponge *Dendrilla nigra*. FEMS Microbiol. Ecol..

[23] Thakur N.L., Muller W.E.G. (2004). Biotechnological potential of marine sponges. Curr. Sci..

[24] Undabarrena A., Beltrametti F., Claverias F.P., Gonzalez M., Moore E.R.B., Seeger M., Camara B. (2016). Exploring the diversity and antimicrobial potential of marine actinobacteria from the comau fjord in Northern Patagonia, Chile. Front. Microbiol..

[25] Claverías F.P., Undabarrena A., González M., Seeger M., Cámara B. (2015). Culturable diversity and antimicrobial activity of actinobacteria from marine sediments in Valparaíso bay, Chile. Front. Microbiol..

[26] Undabarrena A., Ugalde J.A., Castro-Nallar E., Seeger M., Cámara B. (2017). Genome sequence of *Streptomyces* sp. H-KF8, a marine Actinobacterium isolated from a northern chilean patagonian fjord. Genome Announc. Am. Soc. Microbiol..

[27] Gregersen T. (1978). Rapid method for distinction of gram-negative from gram-positive Bacteria. Eur. J. Appl. Microbiol. Biotechnol..

[28] Stach J.E.M., Maldonado L.A., Ward A.C., Goodfellow M., Bull A.T. (2003). New primers for the class Actinobacteria: Application to marine and terrestrial environments. Environ. Microbiol..

[29] Lane D., Stackebrandt E., Goodfellow M. (1991). 16S/23S rRNA Sequencing. Nucleic Acid Techniques in Bacterial Systematics.

[30] Guindon S., Dufayard J.F., Lefort V., Anisimova M., Hordijk W., Gascuel O. (2010). New algorithms and methods to estimate maximum-likelihood phylogenies: Assessing the performance of PhyML 3.0. Syst. Biol..

[31] Felsenstein J. (1985). Confidence limits on phylogenies: An approach using the bootstrap. Evolution.

[32] Posada D. (2008). jModelTest: Phylogenetic model averaging. Mol. Biol. Evol..

[33] Tamura K., Stecher G., Peterson D., Filipski A., Kumar S. (2013). MEGA6: Molecular evolutionary genetics analysis version 6.0. Mol. Biol. Evol..

[34] Gontang E.A., Gaude S.P., Fenical W., Jensen P.R. (2010). Sequence-based analysis of secondary-metabolite biosynthesis in marine actinobacteria. Appl. Environ. Microbiol..

[35] Ayuso A., Clark D., González I., Salazar O., Anderson A., Genilloud O. (2005). A novel actinomycete strain de-replication approach based on the diversity of polyketide synthase and nonribosomal peptide synthetase biosynthetic pathways. Appl. Microbiol. Biotechnol..

[36] Ayuso-Sacido A., Genilloud O. (2005). New PCR primers for the screening of NRPS and PKS-I systems in actinomycetes: Detection and distribution of these biosynthetic gene sequences in major taxonomic groups. Microb. Ecol..

[37] Haber M., Ilan M. (2013). Diversity and antibacterial activity of bacteria cultured from Mediterranean *Axinella* spp. sponges. J. Appl. Microbiol..

[38] Lyman J., Fleming R. (1940). Composition of seawater. J. Mar. Res..

[39] De la Cruz M., González I., Parish C.A., Onishi R., Tormo J.R., Martín J., Peláez F., Zink D., El Aouad N., Reyes F. (2017). Production of ramoplanin and ramoplanin analogs by actinomycetes. Front. Microbiol..

[40] Chambers M., Maclean B., Burke R., Amodei D., Ruderman D., Neumann S., Gatto L., Fischer B., Agus D., MacCoss M. (2012). A cross-platform toolkit for mass spectrometry and proteomics. Nat. Biotechnol..

[41] Pluskal T., Castillo S., Villar-briones A., Ore M. (2010). MZmine 2: Modular framework for processing, visualizing, and analyzing mass spectrometry-based molecular profile data. BMC Bioinform..

[42] Forner D., Berrué F., Correa H., Duncan K., Kerr R.G. (2013). Chemical dereplication of marine actinomycetes by liquid chromatography-high resolution mass spectrometry profiling and statistical analysis. Anal. Chim. Acta.

[43] Ward J. (1963). Hierarchical grouping to optimize an objective function. J. Am. Stat. Assoc..

[44] Gagnon K., Chadwell C.D., Norabuena E. (2005). Measuring the onset of locking in the Peru-Chile trench with GPS and acoustic measurements. Nature.

[45] Baltz R.H. (2006). Marcel Faber Roundtable: Is our antibiotic pipeline unproductive because of starvation, constipation or lack of inspiration?. J. Ind. Microbiol. Biotechnol..

[46] Lazzarini A., Cavaletti L., Toppo G., Marinelli F. (2000). Rare genera of actinomycetes as potential producers of new antibiotics. Antonie Van Leeuwenhoek.

[47] Jose P.A., Jebakumar S.R.D. (2013). The evolving role of natural products in drug discovery. Nat. Rev. Drug Discov..

[48] Fenical W. (1993). Chemical studies of marine bacteria: Developing a new resource. Chem. Rev..

[49] Fenical W., Jensen P.R. (2006). Developing a new resource for drug discovery: Marine actinomycete bacteria. Nat. Chem. Biol..

[50] Magarvey N.A., Keller J.M., Bernan V., Dworkin M., Sherman D.H., Magarvey N.A., Keller J.M., Bernan V., Dworkin M., Sherman D.H. (2004). Isolation and characterization of novel marine-derived actinomycete taxa rich in bioactive metabolites. Appl. Environ. Microbiol..

[51] Jensen P.R., Mincer T.J., Williams P.G., Fenical W. (2005). Marine actinomycete diversity and natural product discovery. Antonie Van Leeuwenhoek.

[52] Bredholdt H., Galatenko O.A., Engelhardt K., Fjærvik E., Terekhova L.P., Zotchev S.B. (2007). Rare actinomycete bacteria from the shallow water sediments of the Trondheim fjord, Norway: Isolation, diversity and biological activity. Environ. Microbiol..

[53] Gontang E.A., Fenical W., Jensen P.R. (2007). Phylogenetic diversity of gram-positive bacteria cultured from marine sediments. Appl. Environ. Microbiol..

[54] León J., Liza L., Soto I. (2007). Actinomycetes bioactivos de sedimento marino de la costa central del Perú. Rev. Peru. Biol..

[55] Maldonado L.A., Stach J.E.M., Ward A.C., Bull A.T., Goodfellow M. (2008). Characterisation of *micromonosporae* from aquatic environments using molecular taxonomic methods. Antonie van Leeuwenhoek.

[56] Yuan M., Yu Y., Li H.R., Dong N., Zhang X.H. (2014). Phylogenetic diversity and biological activity of actinobacteria isolated from the Chukchi Shelf marine sediments in the Arctic Ocean. Mar. Drugs.

[57] Hentschel U., Schmid M., Wagner M., Fieseler L., Gernert C., Hacker J. (2001). Isolation and phylogenetic analysis of bacteria with antimicrobial activities from the Mediterranean sponges *Aplysina aerophoba* and *Aplysina cavernicola*. FEMS Microbiol. Ecol..

[58] Abdelmohsen U.R., Pimentel-Elardo S.M., Hanora A., Radwan M., Abou-El-Ela S.H., Ahmed S., Hentschel U. (2010). Isolation, phylogenetic analysis and anti-infective activity screening of marine sponge-associated actinomycetes. Mar. Drugs.

[59] Kim T.K., Garson M.J., Fuerst J.A. (2005). Marine actinomycetes related to the “*Salinospora*” group from the Great Barrier Reef sponge *Pseudoceratina clavata*. Environ. Microbiol..

[60] Li Z.Y., Liu Y. (2006). Marine sponge Craniella austrialiensis-associated bacterial diversity revelation based on 16S rDNA library and biologically active Actinomycetes screening, phylogenetic analysis. Lett. Appl. Microbiol..

[61] Jiang S., Sun W., Chen M., Dai S., Zhang L., Liu Y., Lee K.J., Li X. (2007). Diversity of culturable actinobacteria isolated from marine sponge *Haliclona* sp.. Antonie Van Leeuwenhoek.

[62] Zhang H., Lee Y.K., Zhang W., Lee H.K. (2006). Culturable actinobacteria from the marine sponge *Hymeniacidon perleve*: Isolation and phylogenetic diversity by 16S rRNA gene-RFLP analysis. Antonie Van Leeuwenhoek.

[63] Radjasa O.K., Sabdono A., Junaidi J., Zocchi E. (2007). Richness of secondary metabolites-producing marine bacteria associated with sponge *Haliclona* sp.. Int. J. Pharmacol..

[64] Abdelmohsen U.R., Yang C., Horn H., Hajjar D., Ravasi T., Hentschel U. (2014). Actinomycetes from red sea sponges: Sources for chemical and phylogenetic diversity. Mar. Drugs.

[65] Schneemann I., Nagel K., Kajahn I., Labes A., Wiese J., Imhoff J.F. (2010). Comprehensive investigation of marine actinobacteria associated with the sponge *Halichondria panicea*. Appl. Environ. Microbiol..

[66] Matobole R.M., van Zyl L.J., Parker-Nance S., Davies-Coleman M.T., Trindade M. (2017). Antibacterial activities of bacteria isolated from the marine sponges *Isodictya compressa* and *Higginsia bidentifera* collected from Algoa Bay, South Africa. Mar. Drugs.

[67] Sarmiento-vizcaíno A., Braña A.F., González V., Nava H., Molina A., Llera E., Fiedler H., Rico J.M. (2016). Atmospheric dispersal of bioactive *Streptomyces albidoflavus* strains among terrestrial and marine environments. Microb. Ecol..

[68] Miller S.I. (2016). Antibiotic resistance and regulation of the gram-negative bacterial outer membrane barrier by host innate immune molecules. MBio.

[69] Delcour A.H. (2009). Outer membrane permeability and antibiotic resistance. Biochim Biophys. Acta.

[70] Vandermolen K.M., Raja H.A., El-Elimat T., Oberlies N.H. (2013). Evaluation of culture media for the production of secondary metabolites in a natural products screening program. AMB Express.

[71] Bode H.B., Bethe B., Höfs R., Zeeck A. (2002). Big effects from small changes: Possible ways to explore nature’s chemical diversity. ChemBioChem.

[72] Sánchez S., Chávez A., Forero A., García-Huante Y., Romero A., Sánchez M., Rocha D., Sánchez B., Avalos M., Guzmán-Trampe S. (2010). Carbon source regulation of antibiotic production. J. Antibiot..

[73] Patin N.V., Duncan K.R., Dorrestein P.C., Jensen P.R. (2015). Competitive strategies differentiate closely related species of marine actinobacteria. ISME J..

[74] Kiranmayi M.U., Sudhakar P., Sreenivasulu K., Vijayalakshmi M. (2011). Optimization of culturing conditions for improved production of bioactive metabolites by *Pseudonocardia* sp. VUK-10. Mycobiology.

[75] Huang R., Yi X., Zhou Y., Su X., Peng Y., Gao C. (2014). An Update on 2,5-Diketopiperazines from Marine Organisms. Mar. Drugs.

[76] Komiyama T., Matsuzawa Y., Oki T., Inui T., Takahashi Y., Naganawa H., Takeuchi T., Umezawa H. (1977). Baumycins, new antitumor antibiotics related to daunomycin. J. Antibiot..

[77] Xu Z., Schenk A., Hertweck C. (2007). Molecular analysis of the benastatin biosynthetic pathway and genetic engineering of altered fatty acid-polyketide hybrids. J. Am. Chem. Soc..

[78] Takada K., Ninomiya A., Naruse M., Sun Y., Miyazaki M., Nogi Y., Okada S., Matsunaga S. (2013). Surugamides A–E, Cyclic Octapeptides with Four D-Amino Acid Residues, from a Marine Streptomyces sp.: LC–MS-Aided Inspection of Partial Hydrolysates for the Distinction of d- and l-Amino Acid Residues in the Sequence. J. Org. Chem..

[79] Wang X., Shaaban K., Elshahawi S., Ponomareva L., Sunkara M., Copley G., Hower J., Morris A., Kharel M., Thorson J. (2016). Mullinamides A and B, new cyclopeptides produced by the Ruth Mullins coal mine fire isolate *Streptomyces* sp. RM-27-46. J. Antibiot..

[80] Macintyre L., Zhang T., Viegelmann C., Martinez I.J., Cheng C., Dowdells C., Abdelmohsen U.R., Gernert C., Hentschel U., Edrada-Ebel R.A. (2014). Metabolomic tools for secondary metabolite discovery from marine microbial symbionts. Mar. Drugs.

