# Supplementary material for: Biodiversity of Actinobacteria from the South Pacific and the Assessment of Streptomyces Chemical Diversity with Metabolic Profiling
Source: Mar Drugs. 2017 Sep 11;15(9):286. doi: 10.3390/md15090286 (PMC5618425; doi:10.3390/md15090286)
Supplement: Supplementary file 1 [file marinedrugs-15-00286-s001.pdf]

**Table S1. Characteristics of the marine samples used for isolation of Actinobacteria in this study.**

| Sample | Sampling site | Zone | | Depth | Coordinates |
|---|---|---|---|---|---|
| **C1** | Chañaral de Aceituno Island | South side of The Window | Marine sediment | 31.5 | 29°2’32.27’’S 71°34’28.69’’W |
| **C2** | | The nameless | | 34.1 | 29°1’10.98’’S 71°34’23.43’’W |
| **C3** | | Walls of Neptune | Marine sponge | (31.5) | 29°2’54.42’’S 71°34’28.69’’W |
| **C4** | | The Canon | | (34.1) | 29°1’23.02’’S 71°33’57.85’’W |
| **V1** | Valparaíso | Carvallo Beach | Marine sediment | 0 | 33°1'8.69"S; 71°38'32.38"W |
| **V2** | | | | 0 | 33°1'8.69"S 71°38'32.38"W |
| **V3** | | | | | Undetermined |
| **V4** | | | | | Undetermined |
| **V5** | | Portales Beach | Marine sediment | 0 | 33°2'0.47"S 71°35'42.36"W |
| **V6** | | | | 0 | 33°2'0.47"S 71°35'42.36"W |
| **V7** | | Punta Ángeles Lighthouse | Marine sediment | 28.4 | 33°1’12,21’’S 71°38’56,41’’W |
| **V8** | | | | 19.2 | 33°1’12,21’’S 71°38’56,41’’W |
| **V9** | | | | 10.0 | 33°1’12,21’’S 71°38’56,41’’W |
| **V10** | | Torpederas Beach | Marine sediment | 29.4 | 33°1’11.05’’S 71°38’43.25’’W |
| **V11** | | | | 19.1 | 33°1’11.05’’S 71°38’43.25’’W |
| **V12** | | | | 6.7 | 33°1’11.05’’S 71°38’43.25’’W |
| **I1** | Easter Island | Motu Nui Islet | Marine sediment | 35 | 27°12’01.07”S 109°27’12.30”W |
| **I2** | | | Marine sponge | 23 | 27°12’01.07”S 109°27’12.30”W |
| **I3** | | | | 23 | 27°12’01.07”S 109°27’12.30’’W |
| **I4** | | Ovahe Beach | Marine sediment | 0 | 27°04’26.01”S 109°19’51.01”W |
| **I5** | | Terevaka | Marine sediment | 22 | 27°5’13.55’’S; 109°24’59.58’’W |
| **I6** | | | Marine sponge | 22 | 27°5’13.55’’S; 109°24’59.58’’W |
| **I7** | | | | 2 | 27°5’13.55’’S; 109°24’59.58’’W |
| **I8** | | | | 32 | 27°5’13.55’’S; 109°24’59.58’’W |
| **E1** | Chiloé Island | Quellón | Sea Urchin | | Undetermined |
| **H1** | Huinay | Lilihuapi Island | Marine sediments | 11.3 | 42°20’634’’S; 72°27’429’’W |
| **H2** | | Tambor Waterfall | Marine sediments | 6.1 | 42°24’161’’S; 72°25’235’’W |
| **H3** | | Punta Llonco | Marine sediments | 25.1 | 42°22’ 32’’S; 72°25’ 4’’W |
| **H4** | | Lloncochaigua River mouth | Marine sediments | 0.25 | 42°22’ 37’’S; 72°27’25’’W |
| **G1** | Penas Gulf | | Marine sediment | 70 | 47°53’19,26’’S; 74°33’0’ W |
| **G2** | | | Marine sediment | 850 | 47°59’41’’S; 73°46’39’W’ |

**Appendix B**

**References**


[1] WHO, “Antimicrobial Resistance.,” 2014.

[2] O. Genilloud, “The re-emerging role of microbial natural products in antibiotic discovery,” *Antonie van Leeuwenhoek, Int. J. Gen. Mol. Microbiol.*, vol. 106, no. 1, pp. 173–188, 2014.

[3] D. J. Newman and G. M. Cragg, “Natural Products as Sources of New Drugs from 1981 to 2014,” *J. Nat. Prod.*, vol. 79, no. 3, pp. 629–661, 2016.

[4] I. Joint, M. Mühling, and J. Querellou, “Culturing marine bacteria - An essential prerequisite for biodiscovery: Minireview,” *Microb. Biotechnol.*, vol. 3, no. 5, pp. 564–575, 2010.

[5] P. G. Williams, “Panning for chemical gold: marine bacteria as a source of new therapeutics,” *Trends Biotechnol.*, vol. 27, no. 1, pp. 45–52, 2009.

[6] T. J. Mincer, P. R. Jensen, C. a Kauffman, and W. Fenical, “Widespread and Persistent Populations of a Major New Marine Actinomycete Taxon in Ocean Sediments Widespread and Persistent Populations of a Major New Marine Actinomycete Taxon in Ocean Sediments,” *Society*, vol. 68, no. 10, pp. 5005–5011, 2002.

[7] A. Prieto, L. Villarreal, S. Forschner, A. Bull, J. Stach, D. Smith, D. Rowley, and P. Jensen, “Targeted search for actinomycetes from near-shore and deep sea marine sediments,” *FEMS*, vol. 84, no. 3, pp. 510–518, 2014.

[8] M. Goodfellow and H. P. Fiedler, “A guide to successful bioprospecting: Informed by actinobacterial systematics,” *Antonie van Leeuwenhoek, Int. J. Gen. Mol. Microbiol.*, vol. 98, no. 2, pp. 119–142, 2010.

[9] A. T. Bull, J. E. . Stach, A. C. Ward, and M. Goodfellow, “Marine actinobacteria: perspectives, challenges, future directions,” *Antonie Van Leeuwenhoek*, vol. 87, no. 1, pp. 65–79, 2005.

[10] D. S. Dalisay, D. E. Williams, X. L. Wang, R. Centko, J. Chen, and J. Raymond, “Marine Sediment-Derived Streptomyces Bacteria from British Columbia , Canada Are a Promising Microbiota Resource for the Discovery of Antimicrobial Natural Products,” *PLoS One*, vol. 8, no. 10, pp. 1–14, 2013.

[11] M. Donia and M. T. Hamann, “Marine natural products and their potential applications as anti-infective agents,” *Lancet Infect. Dis.*, vol. 3, no. 6, pp. 338–348, 2003.

[12] K. Duncan, B. Haltli, K. a. Gill, and R. G. Kerr, “Bioprospecting from marine sediments of New Brunswick, Canada: Exploring the relationship between total bacterial diversity and actinobacteria diversity,” *Mar. Drugs*, vol. 12, no. 2, pp. 899–925, 2014.

[13] A. Penesyan, S. Kjelleberg, and S. Egan, "Development of novel drugs from marine surface associated microorganisms," *Mar. Drugs*, vol. 8, no. 3, pp. 438–459, 2010.

[14] J. Vicente, A. Stewart, B. Song, R. T. Hill, and J. L. Wright, "Biodiversity of Actinomycetes Associated with Caribbean Sponges and Their Potential for Natural Product Discovery," *Mar. Biotechnol.*, vol. 15, no. 4, pp. 413–424, 2013.

[15] A. P. Graça, J. Bondoso, H. Gaspar, J. R. Xavier, M. C. Monteiro, M. De La Cruz, D. Oves-Costales, F. Vicente, and O. M. Lage, "Antimicrobial activity of heterotrophic bacterial communities from the marine sponge Erylus discophorus (Astrophorida, Geodiidae)," *PLoS One*, vol. 8, no. 11, 2013.

[16] N. F. Montalvo, N. M. Mohamed, J. J. Enticknap, and R. T. Hill, "Novel actinobacteria from marine sponges," *Antonie van Leeuwenhoek, Int. J. Gen. Mol. Microbiol.*, vol. 87, no. 1, pp. 29–36, 2005.

[17] T. R. a Thomas, D. P. Kavlekar, and P. a. LokaBharathi, "Marine drugs from sponge-microbe association - A review," *Mar. Drugs*, vol. 8, no. 4, pp. 1417–1468, 2010.

[18] S. T. Khan, J. Musarrat, A. a. Alkhedhairy, and S. Kazuo, "Diversity of bacteria and polyketide synthase associated with marine sponge Haliclona sp.," *Ann. Microbiol.*, vol. 64, no. 1, pp. 199–207, 2014.

[19] J. W. Blunt, B. R. Copp, M. H. G. Munro, P. T. Northcote, and M. R. Prinsep, "Marine natural products.," *Nat. Prod. Rep.*, vol. 27, no. 2, pp. 165–237, 2016.

[20] J. W. Blunt, B. R. Copp, M. H. G. Munro, P. T. Northcote, and M. R. Prinsep, "Marine natural products.," *Nat. Prod. Rep.*, vol. 22, no. 1, pp. 15–61, 2015.

[21] A. P. Graça, F. Viana, J. Bondoso, M. I. Correia, L. Gomes, M. Humanes, A. Reis, J. R. Xavier, H. Gaspar, and O. M. Lage, "The antimicrobial activity of heterotrophic bacteria isolated from the marine sponge Erylus deficiens (Astrophorida, Geodiidae)," *Front. Microbiol.*, vol. 6, no. May, 2015.

[22] J. Selvin, S. Joseph, K. R. T. Asha, W. a. Manjusha, V. S. Sangeetha, D. M. Jayaseema, M. C. Antony, and a. J. Denslin Vinitha, "Antibacterial potential of antagonistic Streptomyces sp. isolated from marine sponge Dendrilla nigra," *FEMS Microbiol. Ecol.*, vol. 50, no. 2, pp. 117–122, 2004.

[23] N. L. Thakur and W. E. G. Muller, "Biotechnological potential of marine sponges," *Curr. Sci.*, vol. 86, no. 11, pp. 1506–1512, 2004.

[24] A. Undabarrena, F. Beltrametti, F. P. Claverias, M. Gonzalez, E. R. B. Moore, M. Seeger, and B. Camara, "Exploring the diversity and antimicrobial potential of marine actinobacteria from the comau fjord in Northern Patagonia, Chile," *Front. Microbiol.*, vol. 7, no. JUL, pp. 1–16, 2016.

[25] F. P. Claverías, A. Undabarrena, M. González, M. Seeger, and B. Cámara, "Culturable diversity and antimicrobial activity of Actinobacteria from marine sediments in Valparaíso bay, Chile," *Front. Microbiol.*, vol. 6, no. JUL, pp. 1–11, 2015.

[26] A. Undabarrena, J. A. Ugalde, M. Seeger, and B. Cámara, "-Genomic data mining of the marine actinobacteria *Streptomyces* sp. H-KF8 unveils insights into multi-stress related genes and metabolic pathways involved in antimicrobial synthesis," *PeerJ*, vol. 5, p. e2912, 2017.

[27] A. Undabarrena, J. a. Ugalde, E. Castro-Nallar, M. Seeger, and B. Cámara, "Genome Sequence of Streptomyces sp. H-KF8, a Marine Actinobacterium isolated from a Northern Chilean Patagonian Fjord," *Genome Announc. Am. Soc. Microbiol.*, vol. 5, no. 6, pp. 8–9, 2017.

[28] T. Gregersen, "Rapid Method for Distinction of Gram-Negative from Gram-Positive Bacteria," *Eur. J. Appl. Microbiol. Biotechnol.*, vol. 5, no. 9, pp. 123–127, 1978.

[29] J. E. M. Stach, L. a. Maldonado, A. C. Ward, M. Goodfellow, and A. T. Bull, "New primers for the class Actinobacteria: Application to marine and terrestrial environments," *Environ. Microbiol.*, vol. 5, no. 10, pp. 828–841, 2003.

[30] E. Moore, A. Arnscheidt, A. Krüger, C. Strömpl, and M. Mau, "Simplified protocols for the preparation of genomic DNA from bacterial cultures," *Mol. Microb. Ecol. Man.*, pp. 3–18, 2004.

[31] D. Lane, *Nucleic Acid Techniques in bacterial systematics*. 1991.

[32] M. Haber and M. Ilan, "Diversity and antibacterial activity of bacteria cultured from Mediterranean Axinella spp. sponges," *J. Appl. Microbiol.*, vol. 116, no. 3, pp. 519–532, 2013.

[33] K. Gagnon, C. D. Chadwell, and E. Norabuena, "Measuring the onset of locking in the Peru-Chile trench with GPS and acoustic measurements.," *Nature*, vol. 434, no. 7030, pp. 205–208, 2005.

[34] R. H. Baltz, "Marcel Faber Roundtable: Is our antibiotic pipeline unproductive because of starvation, constipation or lack of inspiration?," *J. Ind. Microbiol. Biotechnol.*, vol. 33, no. 7, pp. 507–513, 2006.

[35] a. Lazzarini, L. Cavaletti, G. Toppo, and F. Marinelli, "Rare genera of actinomycetes as potential producers of new antibiotics," *Antonie van Leeuwenhoek, Int. J. Gen. Mol. Microbiol.*, vol. 78, no. 3–4, pp. 399–405, 2000.

[36] P. A. Jose and S. R. D. Jebakumar, "The evolving role of natural products in drug discovery," *Nat. Rev. Drug Discov.*, vol. 4, no. 3, pp. 206–220, 2013.

[37] W. Fenical, "Chemical studies of marine bacteria: developing a new resource," *Chem. Rev.*, vol. 93, no. 5, pp. 1673–1683, 1993.

[38] W. Fenical and P. R. Jensen, "Developing a new resource for drug discovery: marine actinomycete bacteria.," *Nat. Chem. Biol.*, vol. 2, no. 12, pp. 666–673, 2006.

[39] N. a Magarvey, J. M. Keller, V. Bernan, M. Dworkin, D. H. Sherman, N. a Magarvey, J. M. Keller, V. Bernan, M. Dworkin, and D. H. Sherman, "Isolation and Characterization of Novel Marine-Derived Actinomycete Taxa Rich in Bioactive Metabolites Isolation and Characterization of Novel Marine-Derived Actinomycete Taxa Rich in Bioactive Metabolites †," *Appl. Environ. Microbiol.*, vol. 70, no. 12, pp. 7520–7529, 2004.

[40] P. R. Jensen, T. J. Mincer, P. G. Williams, and W. Fenical, "Marine actinomycete diversity and natural product discovery," *Antonie van Leeuwenhoek, Int. J. Gen. Mol. Microbiol.*, vol. 87, no. 1, pp. 43–48, 2005.

[41] H. Bredholdt, O. a. Galatenko, K. Engelhardt, E. Fjærvik, L. P. Terekhova, and S. B. Zotchev, "Rare actinomycete bacteria from the shallow water sediments of the Trondheim fjord, Norway: Isolation, diversity and biological activity," *Environ. Microbiol.*, vol. 9, no. 11, pp. 2756–2764, 2007.

[42] E. A. Gontang, W. Fenical, and P. R. Jensen, "Phylogenetic diversity of gram-positive bacteria cultured from marine sediments," *Appl. Environ. Microbiol.*, vol. 73, no. 10, pp. 3272–3282, 2007.

[43] J. León, L. Liza, and I. Soto, "Actinomycetes bioactivos de sedimento marino de la costa central del Perú," *Rev. peru Biol.*, vol. 14, no. 2, pp. 259–270, 2007.

[44] L. A. Maldonado, J. E. M. Stach, A. C. Ward, A. T. Bull, and M. Goodfellow, "Characterisation of micromonosporae from aquatic environments using molecular taxonomic methods," *Antonie van Leeuwenhoek, Int. J. Gen. Mol. Microbiol.*, vol. 94, no. 2, pp. 289–298, 2008.

[45] M. Yuan, Y. Yu, H. R. Li, N. Dong, and X. H. Zhang, "Phylogenetic diversity and biological activity of actinobacteria isolated from the chukchi shelf marine sediments in the arctic ocean," *Mar. Drugs*, vol. 12, no. 3, pp. 1281–1297, 2014.

[46] G. Wang, "Diversity and biotechnological potential of the sponge-associated microbial consortia," *J. Ind. Microbiol. Biotechnol.*, vol. 33, no. 7, pp. 545–551, 2006.

[47] U. Hentschel, M. Schmid, M. Wagner, L. Fieseler, C. Gernert, and J. Hacker, "Isolation and phylogenetic analysis of bacteria with antimicrobial activities from the Mediterranean sponges *Aplysina aerophoba* and *Aplysina cavernicola*.," *FEMS Microbiol. Ecol.*, vol. 35, no. 3, pp. 305–312, 2001.

[48] U. R. Abdelmohsen, S. M. Pimentel-Elardo, A. Hanora, M. Radwan, S. H. Abou-El-Ela, S. Ahmed, and U. Hentschel, "Isolation, phylogenetic analysis and anti-infective activity screening of marine sponge-associated actinomycetes," *Mar. Drugs*, vol. 8, no. 3, pp. 399–412, 2010.

[49] T. K. Kim, M. J. Garson, and J. a Fuerst, "Marine actinomycetes related to the '*Salinospora*' group from the Great Barrier Reef sponge *Pseudoceratina clavata*," *Environ. Microbiol.*, vol. 7, p. , 2005.

[50] Z. Y. Li and Y. Liu, "Marine sponge Craniella austrialiensis-associated bacterial diversity revelation based on 16S rDNA library and biologically active Actinomycetes screening, phylogenetic analysis," *Lett. Appl. Microbiol.*, vol. 43, no. 4, pp. 410–416, 2006.

[51] S. Jiang, W. Sun, M. Chen, S. Dai, L. Zhang, Y. Liu, K. J. Lee, and X. Li, "Diversity of culturable actinobacteria isolated from marine sponge Haliclona sp," *Antonie van Leeuwenhoek, Int. J. Gen. Mol. Microbiol.*, vol. 92, no. 4, pp. 405–416, 2007.

[52] H. Zhang, Y. K. Lee, W. Zhang, and H. K. Lee, "Culturable actinobacteria from the marine sponge Hymeniacidon perleve: Isolation and phylogenetic diversity by 16S rRNA gene-RFLP analysis," *Antonie van Leeuwenhoek, Int. J. Gen. Mol. Microbiol.*, vol. 90, no. 2, pp. 159–169, 2006.

[53] E. Z. O. Radjasa, A. Sabdono, J. Zocchi, "Richness of secondary metabolites-producing from marine bacteria associated with spnges.pdf," *Int. J. Pharmacol.*, vol. 3, no. 3, pp. 275–279, 2007.

[54] U. R. Abdelmohsen, C. Yang, H. Horn, D. Hajjar, T. Ravasi, and U. Hentschel, "Actinomycetes from red sea sponges: Sources for chemical and phylogenetic diversity," *Mar. Drugs*, vol. 12, no. 5, pp. 2771–2789, 2014.

[55] I. Schneemann, K. Nagel, I. Kajahn, A. Labes, J. Wiese, and J. F. Imhoff, "Comprehensive investigation of marine actinobacteria associated with the sponge halichondria panicea," *Appl. Environ. Microbiol.*, vol. 76, no. 11, pp. 3702–3714, 2010.

[56] R. M. Matobole, L. J. Van Zyl, S. Parker-nance, M. T. Davies-coleman, and M. Trindade, "Antibacterial Activities of Bacteria Isolated from the Marine Sponges Isodictya compressa and Higginsia bidentifera Collected from Algoa Bay, South Africa," pp. 8–10, 2017.

[57] M. G. Watve, R. Tickoo, M. M. Jog, and B. D. Bhole, "How many antibiotics are produced by the genus Streptomyces?," *Arch. Microbiol.*, vol. 176, no. 5, pp. 386–390, 2001.

[58] R. E. de L. Procópio, I. R. da Silva, M. K. Martins, J. L. de Azevedo, and J. M. de Araújo, "Antibiotics produced by Streptomyces," *Brazilian J. Infect. Dis.*, vol. 16, no. 5, pp. 466–471, 2012.

[59] J. Bérdy, "Thoughts and facts about antibiotics: Where we are now and where we are heading," *J. Antibiot. (Tokyo).*, vol. 65, no. 8, pp. 441–441, 2012.
